# Psychological Capital, College Adaptation, and Internet Addiction: An Analysis Based on Moderated Mediation Model

**DOI:** 10.3389/fpsyt.2021.712964

**Published:** 2021-11-24

**Authors:** Xiangyang Bi, Jun Jin

**Affiliations:** ^1^Department of Sociology, School of Ethnology and Sociology, Minzu University of China, Beijing, China; ^2^Department of Sociology, School of Social Sciences, Tsinghua University, Beijing, China

**Keywords:** psychological capital, internet addiction, moderated mediation model, emotional adaption, learning adaptation, interpersonal adaptation

## Abstract

Using data from a baseline survey of college students, this study examined the possible mechanism by which psychological capital influences college students' internet addiction through the mediation effect of their individual college adaptability. The study constructed a parallel multiple mediation effect model to help understand the effect mechanisms among these factors. The results indicated that psychological capital had a triple effect on internet addiction: (1) Psychological capital had a direct effect of helping reduce college students' internet addiction; (2) emotional, learning, and interpersonal adaptation not only reduced internet addiction directly but also played mediating roles in the relationship between psychological capital and internet addiction; and (3) the mediation effects of emotional and interpersonal adaptation were moderated by psychological capital, leading to two different modes of mediation effects. As a whole, psychological capital imposes a quadratic effect on internet addiction. The campus policy implications of these findings are discussed.

## Introduction

With the widespread popularity of the Internet, computers, smartphones, and other terminal hardware, being networked has become a trend among contemporary college students. Networks provide convenience for college students' learning and life, but the excessive use of networks may also cause problems. Internet addiction (IA) is one such problem. Numerous studies have found that IA is closely correlated with unhealthy mental states (1, 2), academic failure and social isolation (3, 4), and even suicidal ideation (5, 6). In particular, as Berardis et al. (7, 8) pointed, IA might be associated with the development/concurrence of psychiatric disorders especially in a context of an emotional dysregulation.

The concept of IA was first proposed by American psychiatrist Goldberg (9). Young (10) further developed this concept, and defined it as an impulse control disorder and a significant impairment of social and psychological functioning of the individual caused by the excessive use of the Internet. Although IA was not included in DSM-5 due to some doctrinal and clinical disagreements, Internet gaming disorder (IGD) has long been in the DSM-5 appendix as part of IA (11). In practice, this situation has not prevented a large number of country-specific surveys and studies on IA from proliferating [see (12)].

Recently, IA-related problems among youth have become an academic focus. Scholars have widely highlighted the following factors in their empirical studies: (1) psychological factors, such as personality traits [e.g., (13)] and coping styles [e.g., (14)]; (2) social factors, such as social support [e.g., (15)] and family socioeconomic status [e.g., (16)]; (3) demographic characteristics, such as gender [e.g., (17)]; and (4) situational factors, such as class climate [e.g., (18)].

It is somewhat puzzling that few studies have been performed on IA in relation to psychological capital (hereinafter referred to as “PsyCap”). PsyCap is one of the core concepts of the positive psychology movement of recent years. PsyCap refers to psychological resource beyond human capital, social capital, and a positive state of mind, and it can promote personal growth and improve performance in the process of an individual's development. PsyCap is composed of four operational dimensions: self-efficacy, hope, optimism, and tenacity (19). Empirical studies have shown that PsyCap is related to occupational performance, attitude, and behavior in the general occupational population (20) and plays an active role in college students' learning and daily life by improving academic performance (21), relieving psychological pressure (22), and promoting physical and mental health (23).

Logically, high PsyCap should also help college students use networks properly and reduce addiction orientation. Using 300 Chinese college students' data, Shen and Wang (24) found a negative relationship between PsyCap and IA. Their study confirmed that PsyCap had some explanatory power for IA but did not clarify the specific mechanism between them. Simsek and Sali (25) found the same relationship between the two variables among a sample of 211 college students in Turkey. Khera's study on 130 Indian college students had similar findings (26). However, these studies were also unable to clearly explain the relationship mechanism due to the lack of considering the mediating variables in their models.

In this regard, college adaptation (hereinafter referred to as “ColAda”) is a noteworthy concept. As a special form of social adaptation, ColAda refers to the process in which college students' behavior and ideas change regarding life, learning, and social interaction when they live in the new environment of a university campus. College students are prone to psychological discomfort and even adjustment disorder, partly due to their adolescent/early adult psychological development characteristics. Entering a university means escaping from their parents' monitoring and obtaining more freedom of choice. Moreover, with easier access to the internet and the stronger incentive of computer/network usage, college students are more fragile and more vulnerable to pathological internet use (PIU) (27). It is no exaggeration to conclude that the university life facilitates the risk factors of IA [(28–30): 34–35]. Therefore, it is of practical and theoretical significance to study IA from the perspective of ColAda. It can even deduce that IA is a reflection of college maladaptation. However, until now, only limited number of studies explore the relationship between ColAda and IA. In one of the few existing studies on this topic, Lanthier and Windham (31) suggested that there was a negative relationship between network use and college adjustment. Several studies also found that IA was significantly negatively correlated with all dimensions of the ColAda scale among Chinese college students (32–34).

The relationship between PsyCap and ColAda has also been confirmed (35). However, in the current research on the pathogenesis of IA, no study has integrated PsyCap, ColAda, and IA in the same analytical framework and considered the complex causal mechanisms among these factors, such as possible mediation or moderation effects. This study attempts to explore the influence of PsyCap on the IA of college students considering the possible mediating mechanism *via* ColAda.

As described in a recent review article (36), the mechanism of PsyCap has not been very clear and systematically expounded in theory. Besides the direct effect, there are also two ways in relevant empirical research: the buffering effect (the mediating effect between PsyCap and the outcome variable, or the relationship between them moderated by some specific variables) [e.g., (37, 38)] and the moderating effect [e.g., (39, 40)].

In this study, these effects will be taken into consideration. Based on the findings reviewed above and practical considerations, the relationships among variables of IA (Y), PsyCap (X), and ColAda (M) are hypothesized to be the following: H-1: PsyCap (X) is negatively related to IA (Y); H-2: ColAda (M) is negatively related to IA (Y); H-3: ColAda(M) mediates the relationship between PsyCap (X) and IA (Y); H-4: PsyCap (X) moderates the relationship between ColAda (M) and IA (Y). The reason for H-4 is that individuals with high PsyCap may avoid indulging in networks, even in the face of high college maladaptation problems. In the model, the superposition of indirect and moderating effects (through ColAda) reflects the quadratic effect of PsyCap on IA.

**Figure 1:**
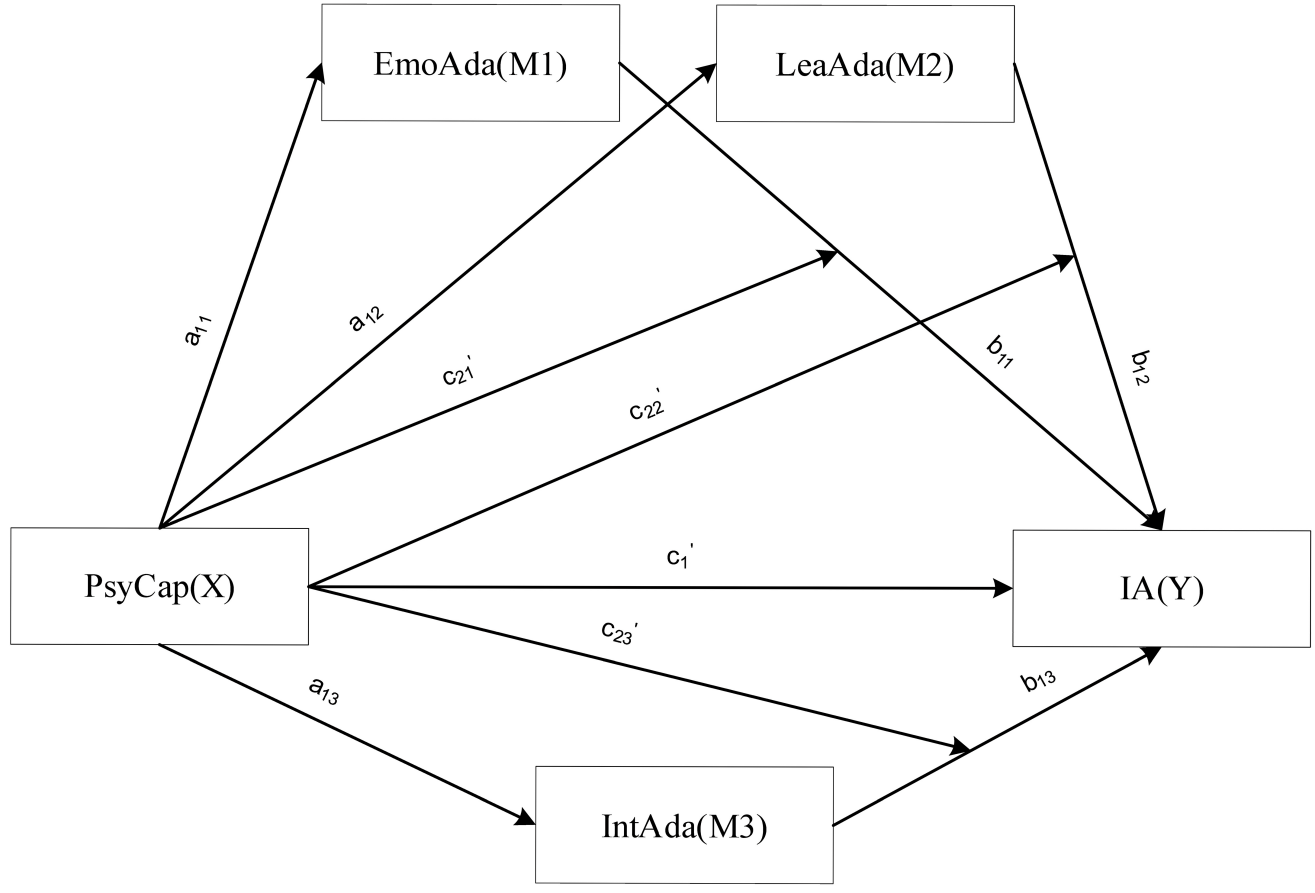
Model diagram of moderated mediation model.

To describe the relationships among all variables according to the above assumptions, this study built a parallel multiple mediation effect model considering the moderation effect of the independent variable, PsyCap (X). The conceptual model is shown in Figure 1^1^.

## Methodology

### Survey and Sample

The data used in this study come from a survey of college students in the China University of Political Science and Law conducted in November 2015^2^. The sample was obtained through random cluster sampling, taking classes as the clusters and students as the elements. Overall, 26.3% of university students were included in the sample. After data cleaning procedures to remove invalid cases, the effective sample size was 2,133^3^. Comparing the structures of the population and the sample, the main relevant indicators of the sample were consistent with the overall structure of the university population (except for a slightly lower proportion of seniors for some practical reasons), indicating good sample representativeness. Because it is inconvenient to apply weights with the bootstrap method, this study did not perform a weighting adjustment for the data.

### Instruments

#### IA (Y)

The Internet Addiction Test (IAT) is most widely used to screen for problematic internet use. The scale, developed by Young (10), is one of the most widely used tools for screening IA. It consists of 20 items. Studies have shown that the scale has good reliability, validity, and unidimensionality (44, 45). This paper used it to measure the level of IA. The Cronbach's α coefficient of the IAT was calculated to be 0.9209 for this sample, indicating a high degree of internal consistency reliability.

#### ColAda (M)

A localized and simplified version of Students Adaptation Adaptability Questionnaire (SACQ) (46, 47) was used to measure the ColAda of college students. The revised scale has 36 items and contains six dimensions including personal–emotional adaptation, learning adaptability, interpersonal adaptation, life adaptation, university satisfaction, and overall assessment. To build a parsimonious model and considering the research topic, this study selected three dimensions and 17 items, i.e., emotional adaptation (M1, 8 items) (hereinafter called “EmoAda”), learning adaptation (M2, 4 items) (hereinafter called “LeaAda”), and interpersonal adaptation (M3, 5 items) (hereinafter called “IntAda”; see Table 1). Items (stared) expressing a negative response were reverse-coded so that a higher total score indicates better adaptation. Based on these data, the Cronbach's α coefficients of three dimensions were calculated to be 0.8012, 0.6836, and 0.7205, respectively, indicating high internal consistency reliability.

**Table 1:** Three dimensions of college adaptation and all items' proportion distribution (%).

	**Very not coincide**	**Not coincide**	**Not certain**	**Coincide**	**Very coincide**
Lately, I have been feeling nervous or anxious*	9.6	32.1	15.0	28.4	14.8
Recently, I have been very depressed*	9.2	26.2	20.0	28.7	15.9
Recently, I have tended to feel tired*	15.9	37.1	17.4	20.9	8.6
Recently, I can't take control of my emotions very well*	6.9	22.5	20.4	30.8	19.4
Recently, my sleep quality has not been good*	11.5	27.2	17.4	23.3	20.6
Sometimes, my mind easily becomes a mess*	12.4	31.1	24.1	20.0	12.4
When dealing with the various pressures at college, I encounter many difficulties*	11.0	32.1	28.9	23.4	4.6
Recently, I have often had a headache*	7.0	11.7	21.0	30.2	30.1
I get along well with my college roommates	3.8	5.1	13.2	36.9	41.0
When in a group of college students, I feel uncomfortable*	2.4	9.6	16.0	35.9	36.1
I find it difficult to get along with people around me*	1.7	9.1	17.0	38.4	33.8
I am afraid to associate with the same or the opposite sex*	2.6	6.7	11.7	32.6	46.4
I keep up with learning	2.4	15.3	29.2	36.6	16.5
I am satisfied with my learning situation	8.4	30.7	30.7	23.6	6.6
I have explicit learning goals	6.1	12.8	28.4	36.3	16.3
I like the profession I am studying to attain	4.6	11.2	25.3	36.7	22.3
I am not interested in the profession I am studying to attain*	3.0	11.0	21.4	29.5	35.0

*Items marked with asterisk are negative statements and the scoring has been redirected before data analysis*.

#### PsyCap (X)

The Positive Psychological Capital Questionnaire (PPQ) (48), a Chinese-localized edition, was used to measure college students' psychological capital. It contains four dimensions including self-efficacy, resilience, hope, and optimism, and 26 items in total. Studies have demonstrated that the co-motivating force of PsyCap is more obvious when regarded as a holistic concept (higher-order factor) (49). Scores on all items in the scale are summed, and a higher score indicates higher psychological capital. The Cronbach's α coefficient of this scale was 0.9038 based on the survey data, showing a high degree of internal consistency reliability.

The variables PsyCap and three dimensions of ColAda were all centered around the grand-mean to make the model estimate more robust, avoid collinearity to some degree, and interpret the output of the model more easily. There are no zero-point responses in these scales in the raw data.

### The Testing Method of Indirect Effect

Indirect effects were tested by a bias-corrected bootstrap method. Because the indirect effect of product terms (ab) is not usually subject to the normal distribution, especially in a small sample, the traditional method of indirect effects, i.e., the Sobel Test (50), might be inaccurate. It is difficult to perform an indirect effect test in some complex models, and the bootstrap method is one of the best ways to do so (51, 52). The bootstrap was set to 1,000 times, and the ML (maximum likelihood) estimation method was used in the process of modeling with M*plus*.

### The Structure of Model

Without a loss of generality, taking the single mediation effect model with the independent variable moderation as an example, the corresponding equations are below (42, 53):


(1) $$\text{M}=a_{0}+a_{1}\text{X}+e_{m}$$

(2) $$\text{Y}=b_{0}+b_{1}\text{M}+c_{1}{}^{\prime }\text{ X}+c_{2}{}^{\prime }\text{ XM}+e_{m}$$

After bringing Equations (1) into (2) and rearranging all terms, the final equation is:


(3) $$\begin{array}{l} \text{Y}=b_{0}+b_{1}(a_{0}+a_{1}\text{X}+e_{m})+c_{1}{}^{\prime }\text{X}+c_{2}{}^{\prime }\text{X}(a_{0}+a_{1}\text{X}+e_{m})\\ \;+e_{y}=(b_{0}+a_{0}b_{1})+[(c_{1}{}^{\prime }+a_{0}c_{2}{}^{\prime })+a_{1}(b_{1}+c_{2}{}^{\prime }\text{X})]\text{X}\\ \;+(b_{1}e_{m}+c_{2}{}^{\prime }Xe_{m}+e_{y}) \end{array}$$

In Equation (3), (*b*_0_+*a*_0_*b*_1_) is the simple intercept, and $\left[(c_{1}{}^{\prime }+a_{0}c_{2}{}^{\prime })+a_{1}(b_{1}+c_{2}{}^{\prime }\text{X})\right]$ is the simple slope, wherein $\left(c_{1}{}^{\prime }+a_{0}c_{2}{}^{\prime }\right)$ is the simple direct effect and $a_{1}(b_{1}+c_{2}{}^{\prime }\text{X})$ is the simple indirect effect. According to this equation, there is no ordinary linear moderation but a quadratic moderation in the model. The regression coefficient of X to Y is also the linear combination of X.

### Findings

#### Descriptive Statistics

The survey revealed that in accordance with the grading standard (i.e., score 40–60 for mild IA, score 60–80 for moderate IA, and score 80–100 for severe IA), 33.2% of students had mild IA, 9.3% had moderate IA, and 0.6% had severe IA. The statistical descriptions of relevant variables in the model are shown in Table 2.

**Table 2:** Statistical descriptions and relation coefficient matrix.

	**Statistical Descriptions**			**Scale**		**Relation Coefficients**						
	**Mean (Centering)**	**Mean**	**Std**	**Min**	**Max**	**Y**	**X**	**M1**	**M2**	**M3**	**XM1**	**XM2**
IA (Y)		40.832	13.487	20	100	1.000						
PsyCap (X)	0.000	90.364	13.111	26	130	−0.343	1.000					
EmoAda (M1)	0.000	24.635	6.315	8	40	−0.308	0.332	1.000				
LeaAda (M2)	0.000	17.235	3.746	5	25	−0.430	0.435	0.262	1.000			
IntAda (M3)	0.000	16.038	3.014	4	20	−0.435	0.332	0.371	0.381	1.000		
PsyCap × EmoAda (XM1)	27.490		97.162			−0.089	0.112	0.018	0.080	0.032	1.000	
PsyCap × LeaAda (XM2)	21.365		56.157			0.028	0.107	0.082	−0.069	−0.071	0.372	1.000
PsyCap × IntAda (XW3)	13.113		41.136			0.085	−0.032	0.036	−0.078	−0.070	0.488	0.428

#### Moderated Mediation Effect Model

By including PsyCap (X), ColAda (M_i_), and IA (Y) and the interaction terms (XM_i_) between PsyCap (X) and ColAda (M_i_), and by setting covariances among M1, M2, and M3, this study built a multiple mediation effect model with the moderation effect of the independent variable. Relevant indicators in the output suggest that the model fits the data well. Being estimated using M*plus*, the results of the model are shown in Table 3.

**Table 3:** The output of multiple mediation model with IDV moderation.

		**Mediation effect**			
		**EmoAda (M1)**	**LeaAda (M2)**	**IntAda (M3)**	**IA (Y)**
1	**Intercept**				40.752^***^ (0.270)
2	**Direct effect** **(X** 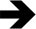 **Y)**				−0.103^***^ (0.024)
3	**—Standardized coefficients**				−0.100^***^ (0.024)
4	**First stage of indirect effect** **(X** 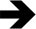 **M)**	0.160^***^ (0.011)	0.124^***^ (0.006)	0.076^***^ (0.005)	
5	**—Standardized coefficients**	0.332^***^ (0.022)	0.435^***^ (0.020)	0.332^***^ (0.021)	
6	**Second stage of indirect effect** **(M** 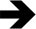 **Y)**	−0.254^***^ (0.048)	−0.874^***^ (0.084)	−1.138^***^ (0.115)	
7	**—Standardized coefficients**	−0.119^***^ (0.022)	−0.244^***^ (0.023)	−0.255^***^ (0.024)	
**Conditional indirect effect** **(X** 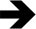 **M** 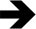 **Y)**					
8	**X** **=** **0**	−0.041^***^ (0.008)	−0.109^***^ (0.011)	−0.087^***^ (0.010)	
9	**Bias-corrected bootstrap 99% confidence interval**	[−0.064, −0.021]	[−0.138, −0.083]	[−0.115, −0.059]	
10	**Interaction Term of XM**	−0.013^***^ (0.003)	0.002 (0.005)	0.030^***^ (0.007)	
**Conditional Indirect Effect (X** 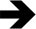 **M** 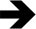 **Y)**					
11	**X** **=** **–SD**	−0.012 (0.010)	−0.113^***^ (0.013)	−0.117^***^ (0.014)	
12	**X** **=** **+SD**	−0.069^***^ (0.011)	−0.105^***^ (0.014)	−0.056^***^ (0.012)	
13	**Pseudo** ***R***^**2**^	0.110^***^ (0.015)	0.189^***^ (0.018)	0.110^***^ (0.014)	0.298^***^ (0.017)
14	**Total indirect effect**				−0.236^***^ (0.017)

*Standard errors in parentheses. Standardized coefficients are StdYX*.

^*^p < 0.05, ^**^p < 0.01, and

*** *p < 0.001*.

*Cov (M1, M2) = 2.767 (0.601), Cov (M2, M3) = 2.669 (0.274), and Cov (M1, M3) = 4.956 (0.469)*.

*LL = −25,711.215, AIC = 51,464.430; CFI = 0.953, RMSEA = 0.073; SRMR = 0.031; N = 2,133*.

##### PsyCap Has a Significant Impact on ColAda

As shown in rows 4 and 5 of Table 3, PsyCap promotes EmoAda, LeaAda, and IntAda. Each of their coefficients is statistically significant (*p* < 0.001, the same below). According to the standardized output, the relationship between PsyCap and LeaAda is the strongest (the standardized path coefficient is 0.435, SE = 0.020). The proportion of variance explained is also slightly higher. The coefficients of the other two mediating variables are nearly equal.

##### All Dimensions of ColAda Have Noticeable Impacts on IA

The relationship between ColAda and IA is statistically significant. Strong adaptation can reduce IA. In other words, maladaptation corresponds to a higher risk of IA. Specifically, the effects of LeaAda and IntAda are higher than those of EmoAda, as revealed by a comparison of the standardized output (row 5 of Table 3). The difference test is statistically significant.

##### ColAda Partially Mediates the Relationship Between PsyCap and IA

ColAda's mediating role is affirmed according to the model output (rows 8 and 9 in Table 3). The three dimensions of ColAda together consist of multiple mediating paths between PsyCap and IA. The mediation effects of EmoAda, LeaAda, and IntAda are all statistically significant. The values are −0.041, −0.109, and −0.087 under the condition that PsyCap is set at the average level. The 99% confidence intervals of the bias-corrected bootstrap method do not include zero points, which indicates the robustness of the results. The difference among three mediation effects is statistically significant. The corresponding test statistic is 0.046 (SE = 0.013). The total indirect effect, i.e., $\sum a_{1i}\left(b_{1i}+{c^{\prime }}_{2i}\text{X}\right)$, is equal to −0.236 and also passes the test of the bias-correction bootstrap method. The percentage of each mediation effect in the total indirect effect is 17.4, 46.2, and 36.9%.

After integrating ColAda as a mediating variable into the model, the direct effect between PsyCap and IA, i.e., ${c^{\prime }}_{1}+\sum a_{0i}c_{2i}^{\prime }$ remains significant. Each value of ColAda is centralized, so *a*_0*i*_ = 0, and the direct effect is just ${c^{\prime }}_{1}$. The value is −0.103 and is statistically significant, indicating that ColAda plays only a partial mediating role in this model. The total effect is −0.103–0.236 = −0.339, in which the indirect effect accounts for 69.6%. The result indicates that in increasing the score by a unit of 1 score relative to the average level, PsyCap can directly reduce the IA score of 0.103 and indirectly reduce the IA score of 0.236 through the mediation effect of the three dimensions of ColAda.

##### PsyCap Also Moderates the Relationship Between ColAda and IA

Considering the independent variable (IDV), i.e., PsyCap, as the moderating variable at the same time, the formula for the mediation effect is equal to $a_{1i}(b_{1i}+c_{2i}{}^{\prime }\text{X})$. The existence of X in the formula reflects PsyCap as the IDV also moderates the relationship between ColAda and IA, i.e., the second stage of the indirect effect. According to the corresponding *p*-value of the coefficients of the interaction terms (XM_i_) between the mediating variables and IDV, it can be inferred that PsyCap is also a moderating variable that influences the relationships among EmoAda, IntAda, and IA separately, but is not a moderator for LeaAda and IA.

The moderation effects of the three dimensions of the ColAda are demonstrated in the coefficients of row 8 in Table 3. In this case, they are actually the conditional indirect effects under the condition that PsyCap is equal to zero. When PsyCap, as a continuous variable, takes two values separately, i.e., plus or minus one standard deviation (±13.111), the corresponding conditional mediation effects of EmoAda, LeaAda, and IntAda can be calculated separately. They are presented in rows 11 and 12 of Table 3. Overall, all of the effects are statistically significant, because none of their 99% bias-corrected confidence intervals includes zero points, which indicates that the results are highly robust^4^. According to the results of this model, because the relationship between LeaAda and IA is not moderated by PsyCap, all conditional indirect effects through LeaAda are close in value^5^.

**Figure 2:**
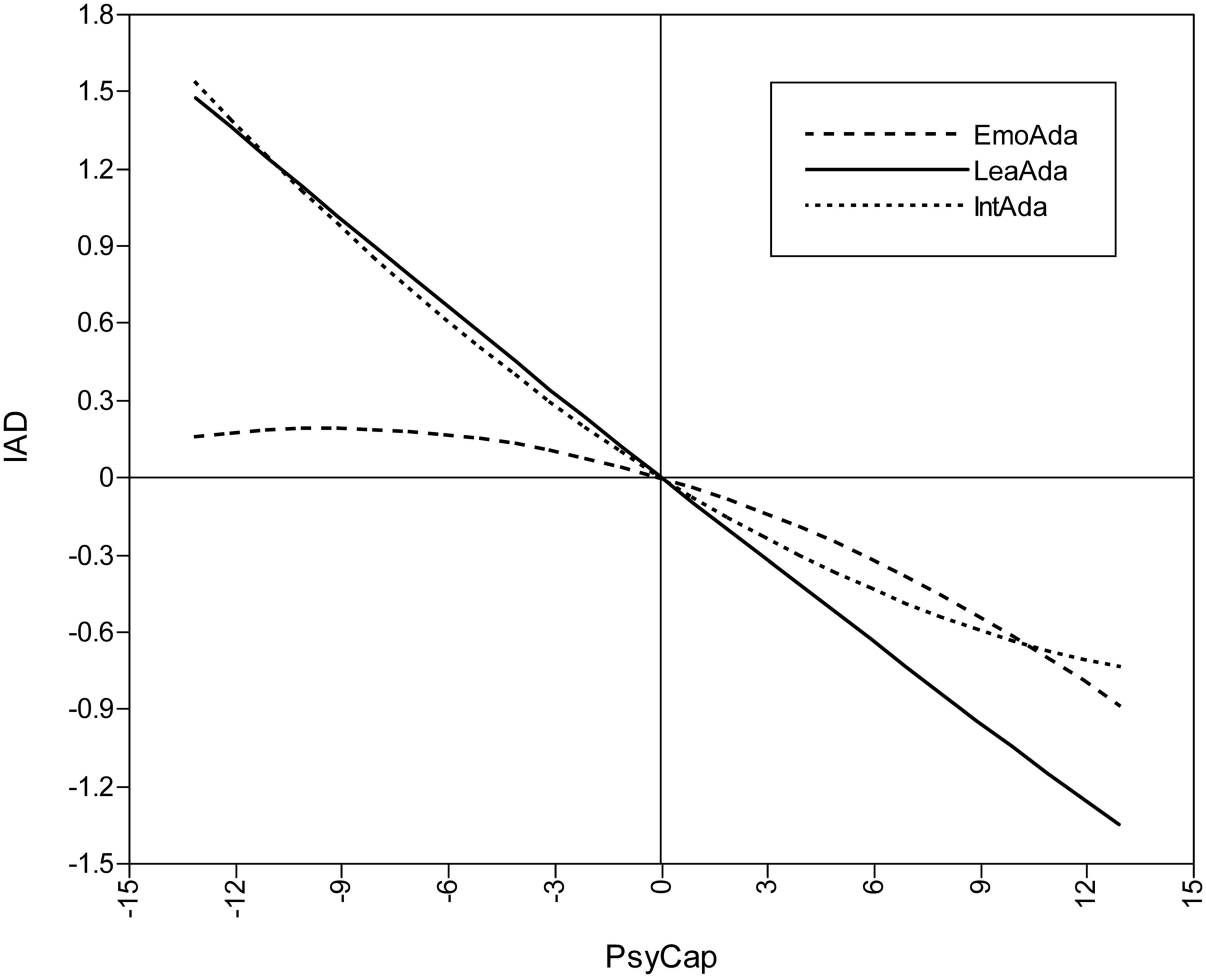
Moderated mediation effect of PsyCap on IA.

**Figure 3:**
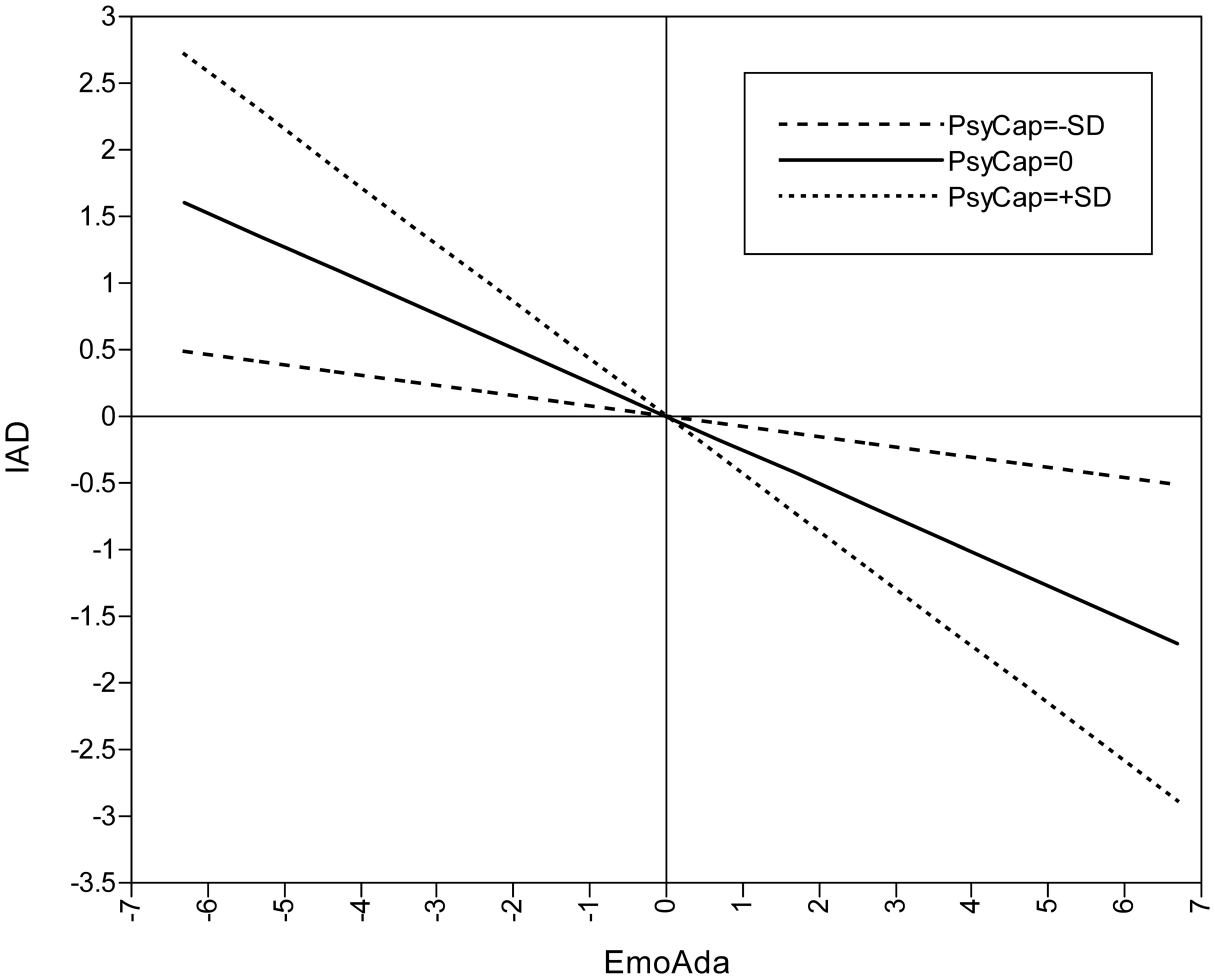
Moderated effect of PsyCap on EmoAda and IA.

**Figure 4:**
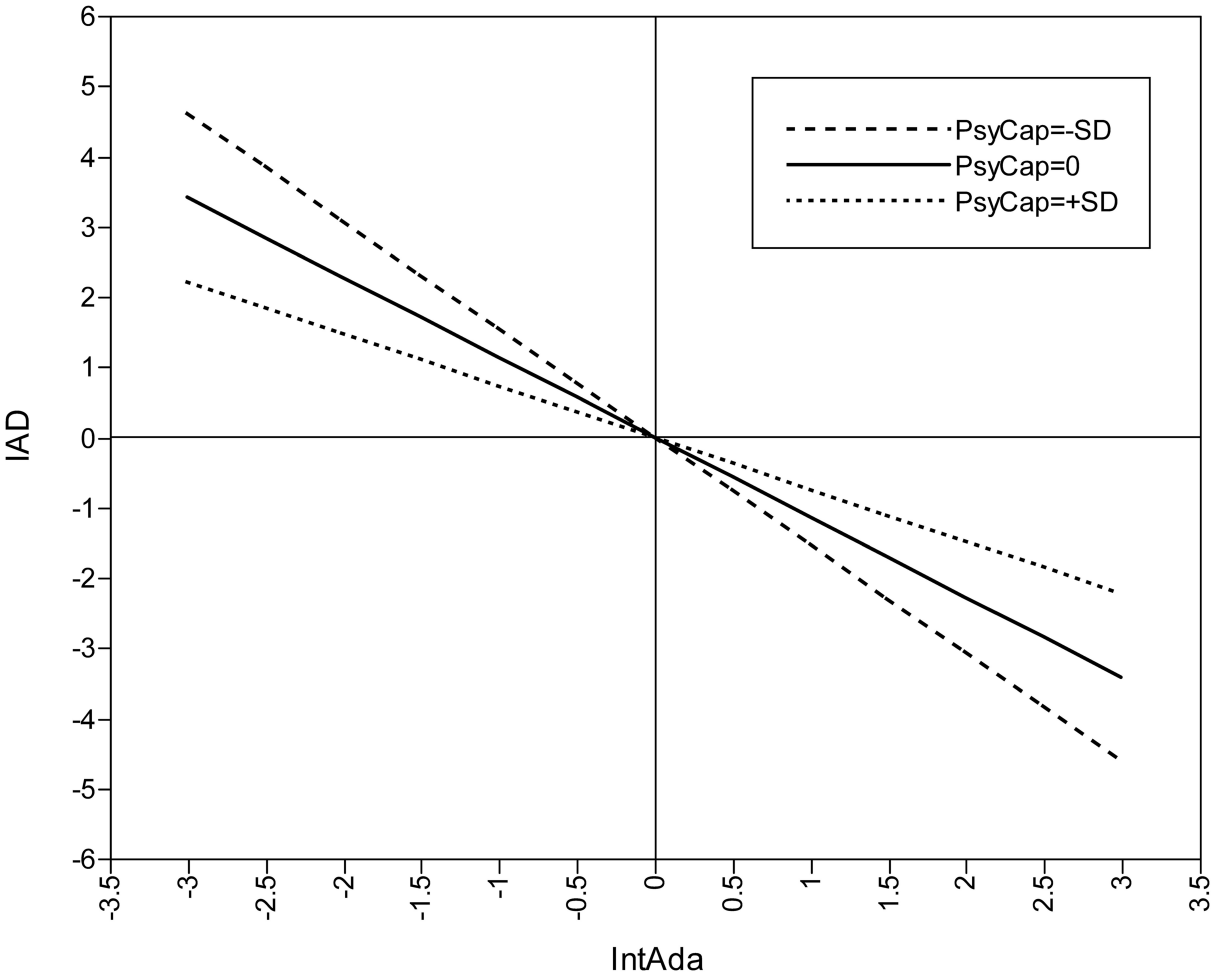
Moderated effect of PsyCap on IntAda and IA.

The conditional mediation effects of the three dimensions of ColAda, as well as the relationships among EmoAda, IntAda, and IA individually moderated by PsyCap (horizontal axis takes ±SD), are shown in Figures 2–4,^6^. Figure 2, referring to results of Table 3, reveals that, in general, an increase in PsyCap diminishes the risk of IA indirectly through the mediation effect of ColAda, but there are different modes in each path.

Since not being moderated by PsyCap in the second stage of this model, the relationship between LeaAda and IA is linear, which means the indirect effect of PsyCap reducing IA *via* LeaAda has stable mode for students as a whole. The flip side of that is LeaAda can invariably influence IA in this causal path, or partially mediate the effect of PsyCap on IA on its own.

By contrast, the effects that PsyCap imposes on IA through the other two dimensions of ColAda are characterized by two different quadratic curves when PsyCap plays moderating roles simultaneously. Specifically, among college students as a whole, the indirect effect *via* EmoAda changes the effect from being smooth to being accelerated. Figure 2 shows that the protective effect against IA is not apparent when PsyCap is low, but it becomes more obvious in the group with high PsyCap as PsyCap increases, which suggests that it is relatively difficult to reduce IA by adjusting EmoAda in college students with low PsyCap. The mode is just the opposite for the mediation effect of IntAda. In the group with low PsyCap, along with the increase in PsyCap, the indirect effect *via* IntAda becomes more distinct. However, in the group with high PsyCap, the antagonism of PsyCap against IA is flattened. That is, in a relative sense, even for the group with low PsyCap, better IntAda can also reduce IA substantially.

Figures 3, 4 also reveal moderation effects of PsyCap on the second stage of the mediating process; that is, the respective relationships between EmoAda, IntAda, and IA. According to these figures, in the group with high PsyCap, the effect of EmoAda on reducing IA is more observable, and for IntAda, the situation is the opposite. Relative to the group with high PsyCap, in the group with low PsyCap, along with the rising of level of IntAda, it is more obvious that IA is reduced. It thus seems that good IntAda can help offset the disadvantage of low PsyCap for reducing college students' IA.

## Conclusion and Discussion

Consistent with the findings of several previous studies, this study confirmed the negative relationships between PsyCap and IA (24) and the relationship pathways among PsyCap, ColAda, and IA (31, 32, 35).

Additionally, this study built a moderated multiple mediation effect model and found that when taking ColAda as mediating mechanism, PsyCap plays a triple role in the relationship with IA: (1) PsyCap has a direct effect on IA (of course, it does not exclude the possibility of isolating other mediating factors within it). (2) Through EmoAda, LeaAda, and IntAda, PsyCap also indirectly influences IA. A high level of PsyCap, through the mediation effect of ColAda, helps reduce the risk of IA. (3) PsyCap moderates the relationships among EmoAda, IntAda, and IA, respectively. For college students as a whole, LeaAda constitutes an invariable factor to reduce IA, and it is not subject to the moderation of PsyCap.

On the whole, PsyCap imposes a quadratic effect on IA. In other words, even not considering the direct effects, psychological capital not only influences certain other factors and thus indirectly affects IA, but also moderates the relationship between these factors and IA. The result echoes the findings described above, namely, the buffering and moderating effects that psychological capital has. The above results provide some clues to IA interventions for the college students population from a positive psychology perspective.

For college students, emotional and interpersonal maladjustment are critical risk factors for IA. The relationship between emotional patterns and IA has been identified, and high emotional stability and emotional intelligence (EI) can be helpful to decrease IA (54). Taking college students as a whole, this study found that reducing IA through emotional adjustment is conditional; it is dependent on the basic psychological qualities of the individual. Relatively speaking, a good EmoAda has a more pronounced effect against IA in the group with high PsyCap.

Moreover, it is the conclusion of many studies that social dysfunction as well as related loneliness, shyness, and other symptoms are important predictors of IA (55–57). The current study illustrates that keeping PsyCap unchanged, good IntAda indeed corresponds to a lower IA level, the protective function of IntAda against IA is higher, and the improvement is more evident even in the group with low psychological quality.

Confronting the widespread problem of IA among college students, these results suggest that psychological and/or social work interventions can start with the cultivation of positive PsyCap (i.e., PCI) and focus on enhancing the emotional, learning, and interpersonal adaptation of this population [(58): 213–216; (59)]. In the short term, assuming that PsyCap is relatively stable, a differentiation strategy should be adopted in the fight against IA: In a holistic sense, for college students with high PsyCap, the emotional adjustment intervention mode can be more well-targeted, while for those with low PsyCap, social skills training interventions are more suitable, as suggested by relevant evaluation studies (60, 61).

Finally, it is important to stress some limitations of this study. First of all, the data used for this study were collected from college students of a university, who were not assessed for any previous or current addictive or psychiatric disorders, or their family history, other than indicators of psychological capital, IA, school adjustment, etc. Further research will need to introduce and control these factors and more background variables to test the robustness of the model, form a more formal theoretical overview of PsyCap and IA, and design some experiments to evaluate the effects of emotional and social interaction interventions.

## Data Availability Statement

The raw data supporting the conclusions of this article will be made available by the authors, without undue reservation.

## Ethics Statement

The studies involving human participants were reviewed and approved by China Communist Youth League Committee at China University of Political Science and Law. Written informed consent for participation was not required for this study in accordance with the national legislation and the institutional requirements.

## Author Contributions

JJ: conceptualization, methodology, writing–review and editing, and supervision. XB: design of study, investigation, data analysis, and writing–original draft. Both authors contributed to the article and approved the submitted version.

## Conflict of Interest

The authors declare that the research was conducted in the absence of any commercial or financial relationships that could be construed as a potential conflict of interest.

## Publisher's Note

All claims expressed in this article are solely those of the authors and do not necessarily represent those of their affiliated organizations, or those of the publisher, the editors and the reviewers. Any product that may be evaluated in this article, or claim that may be made by its manufacturer, is not guaranteed or endorsed by the publisher.

## Footnotes

^1^This is a multiple mediation effect model with the independent variable moderation. A simplified form of this model can be found in Hayes [(41): 398] and Stride et al. (42). For practical examples, see (43).

^2^The survey is completely anonymous and based on informed consent, with the Youth League Committee at the university as the executive agency responsible for supervising the process and ensuring that no harm is done to the participants. For details, see the certification and authorization letter submitted to the editorial board.

^3^After data cleaning, the effective sample size meeting the requirements of this analysis was 2,133. Among them, 64.6% were girls and 35.4% were boys; the mean age was 19.6 years, with a standard deviation of 1.3 years; 20.2% were from rural and 79.8% were from urban; 31.6, 23.6, 31.8, and 13.0% were first-year, second-year, third-year, and fourth-year college students, respectively.

^4^Partially, the conditional mediation effect of EmoAda is not statistically significant when PsyCap takes –SD, which suggests that the relationship between EmoAda and IA is not moderated by PsyCap for those students with a low level of EmoAda. However, overall, psychological capital has a moderating effect on the relationship between emotional adaptation and Internet addiction.

^5^In order to test the robustness of this model's results across genders, we have run the model separately for male and female students. The results show that the mediation effect is robust. Moderation effects are largely robust, except for the emotional adaptation dimension, which was not statistically significant among male college students.

^6^All three figures were exported by M*plus* coding. See the relevant attachment.
